# Life course factors associated with metabolically healthy obesity: a protocol for the systematic review of longitudinal studies

**DOI:** 10.1186/s13643-018-0713-x

**Published:** 2018-03-27

**Authors:** E. M. Robson, S. Costa, M. Hamer, W. Johnson

**Affiliations:** 1 School of Sport, Exercise and Health Sciences, Loughborough University, Loughborough, Leicestershire LE11 3TU UK; 2 UKCRC Centre for Diet and Activity Research (CEDAR), MRC Epidemiology Unit, University of Cambridge School of Clinical Medicine, Cambridge, CB2 0QQ UK

**Keywords:** Metabolically healthy obesity, Metabolic syndrome, Obesity, Life course, Longitudinal study, Body size, Body composition, Puberty, Lifestyle behaviours, Psychosocial stress

## Abstract

**Background:**

There is heterogeneity among obese individuals, as some appear to have healthier metabolic profiles and decreased health risks. These individuals are defined as metabolically healthy obese (MHO), whilst those with unhealthy metabolic profiles are defined as metabolically unhealthy obese (MUO). To date, most research on MHO has been cross-sectional or focused on disease prognosis. However, longitudinal studies are required to provide greater insight into the life course factors that contribute to the development of MHO. This study aims to systematically review longitudinal studies investigating the association between life course exposures and future MHO status.

**Methods:**

Electronic databases (MEDLINE, SCOPUS, and Web of Science) will be searched using a trialled search strategy. Studies will be included following a double-screening process according to inclusion criteria to assess eligibility. Studies eligible for inclusion will include those that have a longitudinal observational design where a life course exposure occurred or was measured at least 1 year before the outcome, investigate a human study population, are published in English after 1956, and investigate the association between ≥ 1 life course exposure and ≥ 1 outcome that reflects a measure of cardiometabolic resilience to obesity. Accepted life course exposures will include body size, body composition, pubertal development, smoking, diet, physical activity, sedentary behaviour, and psychosocial stress. The primary measure of cardiometabolic resilience to obesity will be MHO as an outcome (at follow-up). Studies investigating the development of cardiometabolic risk factors in an obese group without specifying MHO will also be accepted, such as the development of the metabolic syndrome (MetS) in an obese group. Key results of included studies will be tabulated, and a narrative synthesis will be conducted.

**Discussion:**

This will be the first systematic review to summarise the literature on the life course correlates of MHO. Importantly, it may highlight which modifiable lifestyle factors could be targeted to delay the onset of cardiometabolic complications among the obese.

**Systematic review registration:**

PROSPERO CRD42017057992

**Electronic supplementary material:**

The online version of this article (10.1186/s13643-018-0713-x) contains supplementary material, which is available to authorized users.

## Background

Obesity, which is most commonly defined as a body mass index (BMI) ≥ 30 kg/m^2^, is a public health problem in both developed and developing countries, with the global age-standardised obesity prevalence estimated to be 25.7% [1]. Obesity and its associated disease outcomes such as type 2 diabetes mellitus (T2DM), cancer, and cardiovascular disease (CVD) pose an individual and economic burden worldwide. It is estimated that by 2030, the costs due to preventable diseases associated with obesity will cost the United Kingdom (UK) £26 billion and the Unites States of America (USA) $66 billion annually [2].

However, obese individuals do not form a single homogenous group. Research has demonstrated that a subset of obese individuals has healthier metabolic profiles and decreased health risks compared to other obese participants [3]. The concept of metabolically healthy obesity (MHO) and metabolically unhealthy obesity (MUO) has thus been proposed. Metabolically healthy obesity is a condition characterised by obesity in the absence of metabolic abnormalities (e.g. high blood pressure, high blood glucose, adverse lipid profile). On the other hand, metabolically unhealthy obesity (MUO) is characterised by obesity accompanied by metabolic abnormalities. The estimated prevalence of the MHO phenotype varies due to inconsistencies in the criteria used to define MHO [3], but it has been estimated to be in the range of 10–47.7% [4–7].

Much research has looked at the disease prognosis associated with MHO and MUO. It has been proposed that MHO is not a stable condition, rather a transient state before the development of MUO [8]. Studies have demonstrated high rates of transitioning from MHO to MUO, with transitioning rates increasing with longer follow-up periods [8–10]. For example, studies have found over 1 year of follow-up 21.4% of MHO individual’s transition to MUO [9], over eight and a half years of follow-up 44.% of MHO transition to MUO [8], and over 10 years of follow-up 64.7% transition [10].

Further, it is unlikely that MHO is a completely benign condition, rather a condition where the cardiometabolic health risks are intermediate between MUO and metabolically healthy normal weight (MHNW). For example, a meta-analysis of prospective cohort studies found MHO individuals to have approximately four times increased risk (relative risk (RR) = 4.03, 95% confidence interval (CI) 2.66–6.09) and MUO approximately nine times increased risk of developing T2DM (RR = 8.93, 95% CI 6.86–11.62), compared to MHNW [11]. Similarly, another meta-analysis found the same pattern for relative risk of developing CVD [12].

Although research demonstrates that MHO is not benign and may be an intermediate state before the development of MUO, understanding which life course factors are related to MHO is still important [13, 14]. Understanding the life course factors which are related to MHO may inform us of potentially modifiable lifestyle factors which could be the focus of interventions to prevent or delay the development of cardiometabolic disease, even in the presence of obesity.

To date research which has investigated why differences exist between the metabolic health of obese individuals has been largely cross-sectional [15–20]. For example, higher physical activity [15–18], lower sedentary time [17], and smoking [18] have been found to be cross-sectionally associated with the MHO phenotype. In the literature, there appears to be a less longitudinal analysis of factors associated with MHO. Longitudinal analysis of factors associated with MHO would better infer the direction of possible associations and provide a superior estimate of causality, compared to current cross-sectional analyses of such associations. Further, longitudinal analyses are required to elucidate factors across the life course which may predict MHO and could thus be the target of prevention programmes.

To date, no systematic review has summarised the existing longitudinal studies investigating the correlates of MHO. The aim of this study will be to systematically review the literature on the life course correlates of MHO, focusing on body size, body composition, pubertal development, lifestyle behaviours, and psychosocial stress.

## Methods

### Registration

This study protocol is registered with the PROSPERO database (registration number: CRD42017057992).

### Search methods

Three medical databases will be searched; these include MEDLINE, Scopus, and Web of Science. Studies will be searched for using the EBSCO interface for the MEDLINE database, the Elsevier interface for the Scopus database, and the Thomson Reuters interface for the Web of Science database. The search for eligible studies will be carried out in a systematic manner, using synonym free-text words to identify eligible articles until the date of the last search. Truncation commands, Boolean logic, and proximity operators will be used when carrying out the searches and adapted in line with the interface used, see Table 1 for search tools and techniques to be used.

**Table 1:** Tools and techniques that will be used in the online database search

Tool/technique	Description	Example
Capturing phrases	(phrase) used in MEDLINE database searching and “phrase” used in Scopus and Web of Science databases.	(body size trajector*) “body size trajector*”
Boolean logic operators	‘AND’ to combine searches, so databases search for the combination instead of singular search fields.	*Search field 1 AND search field 2 AND search field 3*
	‘OR’ will be used to instruct the database to only retrieve results with at least one of the search terms.	Smok* OR diet* OR alcohol* OR ‘physical activit*’ OR exercis* OR fit* OR psychosocial OR stress*
Proximity operators	NEAR/3 will be used when using Web of Science, W/3 when using Scopus, and N3 when using MEDLINE. These proximity operators instruct the database to only retrieve 2 or more words if they are within 3 words of each other.	Metabolic* NEAR/3 health* NEAR/3 obes* Metabolic* W/3 health* W/3 obes* Metabolic* N/3 health N/3 obes*
Truncation commands	‘root word*’ the asterisks is inputted where the root word naturally finishes, and this instructs the search engine to capture all possible variations of the root word, by searching all the possible word endings.	Exercis* will capture: exercise, excercising, exercised
Restrictions	Dependent upon the search engine used, filters can be used to restrict searches.	MEDLINE via EBSCO: *Date of publication 1960-2017* *Source Type: Academic journals* Scopus via Elsevier *English Language* *Date of publication 1960-2017* *Publication type: articles* Web Of Science via Thomas Reuters *English Language* *Date of publication 1946-2017* *Publication type: articles*

The search strategy has been developed using previous literature which has highlighted relevant key themes or strong theoretical rationale for the inclusion of certain themes. Search terms have been discussed between authors, and piloted in a variation of trialled search strategies, to enhance the efficacy of the final search, see Additional files 1, 2, and 3 for the search strategies used.

Search results from the different interfaces will be exported into RefWorks and merged. Following this, RefWorks will be used to search for duplicates, using a combination of automatic and manual processes. Firstly, the ‘Search for duplicates’ command will be used, and identified duplicates will be removed. Secondly, the remaining records will be manually screened for possible remaining duplicates, and any identified duplicate records will be eliminated.

### Eligibility criteria

There will be two phases to the selection of studies to be included in the review. In each phase articles will be screened according to the following eligibility criteria outlined in Table 2. Screening of studies will be a semi-automated process using distillerSR® software.

**Table 2:** Eligibility criteria

Eligibility question	Response
• 1) Study population: Is a human study population being investigated?	Yes, No, or Not Clear
• 2) Association**:** Is the study testing the association between ≥ 1 life course exposure (i.e. body size, body composition, pubertal development, lifestyle behaviour, or psychosocial stress) and ≥ 1 measure of cardiometabolic resilience to obesity as an outcome?	Yes, No, or Not Clear
• 3) Study design: Is it a longitudinal observational design where the life course exposure occurred or was measured ≥ 1 year before the outcome?	Yes, No, or Not Clear

#### Exposure(s)

Life course exposures will include body size (e.g. body size trajectories, BMI, waist-to-hip ratio (WHR), height), body composition (e.g. muscle mass, fat mass, visceral fat), pubertal development (e.g. age of onset of puberty, tanner stages, menarche, genetic variants for puberty), lifestyle behaviours (e.g. smoking, physical activity, sedentary behaviour, diet), and psychosocial stress measures (e.g. adversity, maltreatment, anxiety, depression, socioeconomic status, social occupational class, income, education and measures of the stress response, e.g. diurnal cortisol rhythms). To be included, exposures can be measured at any point in the participants’ lifetime, but this must be before the outcome of cardiometabolic resilience to obesity has been measured. The exposure measurements can be self-reported or measured directly by researchers or medical staff.

#### Outcome(s)

Studies will be included if they have measured cardiometabolic resilience to obesity as an outcome. Cardiometabolic resilience in the present study is defined as an obese individual’s resilience to the typical cardiometabolic complications that accompany carrying excess weight, for example, the development of unfavourable metabolic profiles, such as elevated blood pressure, abnormal lipid profiles, impaired glucose metabolism, and systemic inflammation [21].

The primary measure of cardiometabolic resilience to obesity will be MHO. To date, there is no universal consensus of a standard definition to diagnose MHO [22]. Some studies use obesity accompanied by the presence or absence of metabolic syndrome (MetS) to diagnose MUO or MHO, whilst some studies use the presence or absence of selected cardiometabolic risk factors, and other studies additionally incorporate the presence or absence of inflammatory markers into their definitions [22]. Therefore, the present study will accept any method of diagnosing MHO, due to the lack of universal consensus on a definition. Other terms used to describe MHO or MUO such as cardiometabolic/metabolic health/unhealthy obese/obesity, cardiometabolic/metabolic abnormal/normal obese/obesity, and cardiometabolic/metabolic benign/at-risk obesity will be accepted as a measure of MHO.

In addition, there are studies where the development of cardiometabolic risk factors is investigated in obese individuals, but the metabolic status is not specifically defined or implied as being ‘healthy’ or ‘unhealthy’; these studies will also be included, for example, studies investigating the presence or absence of MetS in an obese group, where MHO/MUO is not defined specifically.

### Selection of studies

There will be two phases to the selection of studies to be included in the review. In each phase, articles will be screened according to the following eligibility criteria:

#### Phase 1

Following retrieval of articles found via database searching, title and abstracts of these articles will be screened by a primary reviewer (ER) according to the eligibility criteria. Articles with responses ‘yes’ or ‘not clear’ to all three eligibility criteria checkpoints will pass to phase 2, if not, they will be excluded.

#### Phase 2

The full texts for potentially eligible articles found via database searching will be obtained and screened by the primary reviewer (ER) against eligibility criteria. This will be carried out. If responses to all four eligibility criteria checkpoints are ‘yes’, the study will be included in the systematic review. If any of the responses are ‘not clear’, the study in question will be discussed with the senior author and a decision whether to include or exclude the study will be made. If any of the responses is ‘no’, the study will be excluded and the reason(s) noted.

Following this, a random 10% of the full texts screened in phase 2 will be double-screened by a secondary reviewer (WJ) and compared against the same 10% screened by the primary reviewer. If any discrepancies occur between the two reviewers in the decision to include or exclude articles in the 10% of randomly selected studies, they will be resolved by the team and recorded.

### Data extraction and management

Data to be extracted will include data on the citation, study design, participant, exposure(s), outcome(s), and statistical analyses. This data will be extracted by the primary reviewer and inputted into data extraction forms (see Additional file 4). If any doubt arises during the extraction of this information or what to extract, it will be discussed with the secondary reviewer. If any uncertainty arises, it will be recorded.

### Quality assessment

The Newcastle-Ottawa Quality Assessment Scale [23] for cohort studies will be used to assess the quality of included studies and modified where necessary, to suit the nature of the studies included in the review. Modifications will be made to ensure that the three most fundamental domains are being assessed, which include appropriate selection of participants, measurement of variables, and control of confounding [24], see Additional file 5 for the modified scale that will be used. Quality assessment will be a manual process. Data will be extracted and inputted into quality assessment forms and a score calculated (see Additional file 6); these scores will then be reported. Three main domains will be assessed: selection, comparability, and outcome. Selection domain assesses the representativeness of the cohort, whether the sample used in the analysis is representative of the initial cohort, ascertainment of biological exposures, and whether there is a demonstration of presence or absence of the outcome at the start of the study. Comparability assesses whether studies have controlled for confounders in their main analyses. Outcome assesses ascertainment of the outcome, whether the follow-up period was long enough for outcomes to occur and whether the follow-up of cohorts was adequate.

Quality assessment will be carried out primarily by reviewer ER, and secondary reviewer WJ will assess 10% of the included studies. Any discrepancies between quality assessment scored by the two reviewers will be noted, discussed, and resolved.

### Strategy for synthesis

Key information on characteristics, results, and quality scores of included studies will be tabulated (see Additional files 4 and 6). Following this, a narrative synthesis will be conducted. The review will not include a meta-analysis because we are not summarising literature on a single or a few simple associations. Our search strategy allows for multiple exposures, outcomes, and study designs, thereby making an a priori decision to conduct a meta-analysis of results unjustified.

Firstly, in the narrative review, the number of studies to be included in the synthesis will be reported, and characteristics of studies will be described, including the location and study population.

Secondly, the narrative synthesis will report and discuss the methods used to define primary (MHO) and secondary outcomes (MetS in an obese group), and the quality of the methods used will be critiqued.

Finally, associations of life course factors and primary (MHO) and secondary outcomes (MetS in an obese group) will be explored. The findings of studies will be grouped according to exposure type: body size (e.g. body size trajectories, BMI, WHR, height), body composition (e.g. muscle mass, fat mass, visceral fat), pubertal development (e.g. age of onset of puberty, tanner stages, menarche, genetic variants for puberty), lifestyle behaviours (smoking, physical activity, sedentary behaviour, diet), and psychosocial stress exposures (e.g. adversity, maltreatment, anxiety, depression, socioeconomic status, social occupational class, income, education, and measures of the stress response). Within this, similarities and differences of findings will be reported, the strength of findings will be reflected upon, and between-study heterogeneity will be evaluated.

### Amendments to protocol

Any substantial changes to the protocol will be registered with PROSPERO and will be documented in the final publication of the systematic review.

### Reporting

This protocol is being reported according to the ‘Preferred Reporting Items for Systematic Reviews and Meta-analysis Protocols (PRISMA-P) 2015 statement’ [25], see Additional file 7 for the PRISMA-P 2015 checklist. The systematic review and findings will be reported according to the ‘Preferred Reporting Items for Systematic Reviews and Meta-Analyses: the PRISMA statement (2009)’ [26].

### Dissemination

The review will be published in an international, peer-reviewed journal. Further, results will be presented to the research community and wider public, via a number of academic and non-academic outlets. For example, results will be presented at relevant academic conferences, and results will be summarised and shared via social media channels.

## Discussion

This systematic review will be the first to summarise the literature on the life course factors associated with MHO. The relationship between different exposures across the lifetime and MHO or MUO outcome and features of between-study heterogeneity will be explored.

This information will be important in understanding what we currently know on the subject of cardiometabolic resilience to obesity and identifying gaps for future research. In particular, results of the study offer the potential to highlight which modifiable lifestyle factors could be targeted in prevention, intervention, or treatment programmes to delay the onset of cardiometabolic complications among the obese. Helping to improve the health outcomes of the obese is of particular significance considering the high rates of obesity globally [1], and the financial burden placed upon countries healthcare systems, in part due to the cardiometabolic complications associated with overweight and obesity [2].

## Additional files

Additional file 1: **Figure S1.** Search strategy (MEDLINE via EBSCO). (PDF 119 kb)

Additional file 2: **Figure S2.** Search strategy (Scopus via ELSEVIER). (PDF 119 kb)

Additional file 3: **Figure S3.** Search strategy (Web Of Science via Thomson Reuters) (PDF 119 kb)

Additional file 4: Data extraction form. (PDF 21 kb)

Additional file 5: **Figure S4.** Adapted Newcastle-Ottawa Quality Assessment Scale for cohort studies (PDF 774 kb)

Additional file 6: Quality assessment data extraction form. (PDF 231 kb)

Additional file 7: PRISMA-P 2015 checklist. (PDF 346 kb)

## Abbreviations

AUC: Area under the curve
BMI: Body mass index
CAR: Cortisol awakening response
CEDAR: Centre for diet and activity research
CVD: Cardiovascular disease
MetS: Metabolic syndrome
MHNW: Metabolically healthy normal weight
MHO: Metabolically healthy obese/obesity
MUO: Metabolically unhealthy obese/obesity
PRISMA-P: Preferred reporting items for systematic reviews and meta-analysis protocols
RR: Relative risk
T2DM: Type 2 diabetes mellitus
UK: United Kingdom
USA: United States of America
WHR: Waist-to-hip ratio
