# Supplementary material for: Life course factors associated with metabolically healthy obesity: a protocol for the systematic review of longitudinal studies
Source: Syst Rev. 2018 Mar 27;7:50. doi: 10.1186/s13643-018-0713-x (PMC5870377; doi:10.1186/s13643-018-0713-x)
Supplement: Supplementary file 1 — Figure S1. Search strategy (MEDLINE via EBSCO). (PDF 119 kb) [file 13643_2018_713_MOESM1_ESM.pdf]

Additional file 1

Figure S1. Search Strategy, MEDLINE via EBSCO

1. metabolic*
2. cardiometabolic*
3. health*
4. unhealth*
5. normal
6. abnormal
7. benign
8. “at-risk”
9. obes*
10. MHO
11. “BMI”
12. “WHR”
13. “waist circumference”
14. “body composition”
15. abdominal
16. visceral
17. adipos*
18. fat*
19. muscle
20. weight
21. height
22. “body size trajector*”
23. “birth weight”
24. birthweight
25. growth
26. “early onset obes*”
27. pubert*
28. menarch*
29. smok*
30. diet*
31. alcohol*

32. “physical activit*”
33. exercis*
34. fit*
35. sedentary
36. psychosocial
37. stress*
38. adversity
39. adverse experience*
40. maltreatment
41. anxiety
42. depress*
43. socioeconomic*
44. socio-economic*
45. social occupation* class
46. income
47. education
48. cortisol
49. Limit to English Language
50. Limit to publications between 1960-2017
51. 1 or 2 N3 3 or 4 or 5 or 6 or 7 or 8 N3 9 (Search Title, Abstract, Key words)
52. 9 and 10 (Search Title, Abstract, Key words)
53. 11 or 12 or 13 or 14 or 15 or 16 or 17 or 18 or 19 or 20 or 21 or 22 or 23 or 24 or 25 or 26 or 27 or 28 or 29 or 30 or 31 or 32 or 33 or 34 or 35 or 36 or 37 or 38 or 39 or 40 or 41 or 42 or 43 or 44 or 45 or 46 or 47 or 48 (Field searched (Field searched Title, Abstract, Key words)
54. 51 or 52
55. 49 and 50
56. 53 and 54 and 55

Figure 1 *represents the search strategy used, whereby 1- 48 represent all the key-words used in the search, 51-53 represent how the key words were organised into separate fields, 54 represents how the separate were fields were merged to create another search field, 55 represents the restrictions grouped, and 56 represents how*

the separate fields were combined to formulate the final search in addition to the restrictions that were selected when running the search.
