# Supplementary material for: Life course factors associated with metabolically healthy obesity: a protocol for the systematic review of longitudinal studies
Source: Syst Rev. 2018 Mar 27;7:50. doi: 10.1186/s13643-018-0713-x (PMC5870377; doi:10.1186/s13643-018-0713-x)
Supplement: Supplementary file 4 — Data extraction form. (PDF 21 kb) [file 13643_2018_713_MOESM4_ESM.pdf]

Additional file 4. Data extraction form

Citation Details<br>• Title<br>• Year of publication<br>• Authors<br>• Study ID<br>• Pubmed ID<br>• Country of publication	Study Details<br>• Design<br>• Time<br>• Duration<br>• Setting<br>• Sample size	Participants details<br>• Sample size<br>• Age range<br>• % male and female<br>• Ethnicity<br>• % drop out	Exposure(s) variable details<br>• Exposure type<br>• Measurement type<br>• Time(s) and number of measurements<br>• Categorisation criteria (if applicable)	Outcome(s) Variable details<br>• Exposure type<br>• Measurement type<br>• Time(s) and number of measurements<br>• Categorisation criteria (if applicable)	Statistical analyses<br>• Analysis used<br>• Association<br>• Covariates adjusted for<br>• Mediating factors<br>• Results
