# Supplementary material for: Life course factors associated with metabolically healthy obesity: a protocol for the systematic review of longitudinal studies
Source: Syst Rev. 2018 Mar 27;7:50. doi: 10.1186/s13643-018-0713-x (PMC5870377; doi:10.1186/s13643-018-0713-x)
Supplement: Supplementary file 5 — Figure S4. Adapted Newcastle-Ottawa Quality Assessment Scale for cohort studies (PDF 774 kb) [file 13643_2018_713_MOESM5_ESM.pdf]

Additional file 5. Figure S4

Newcastle-Ottawa Quality Assessment for cohort studies (Adapted)

A. Selection

1) Representativeness of the cohort
   a) Truly representative of the source population (***one star***)
   b) Somewhat representative of the source population (***one star***)
   c) Selected group of users e.g nurses, volunteers
   d) No description of the derivation of the cohort
   e) Not clear

2) Is the analysis sample representative of the full cohort?
   a) Yes – information given (***one star***)
   b) Somewhat – information provided, but sample different or no real comparison given
   c) No information given/ not clear

3) Exposure: Ascertainment of biological exposures
   a) Secure record (e.g surgical records) (***one star***)
   b) Directly measured by the study(***one star***)
   c) Doesn't include biological exposures (***one star***)
   d) Mixture of secure record and direct measurement by the study(***one star***)
   e) Structured interview
   f) Written self-report
   g) No description/ not clear

4) Demonstration that the outcome was not present at the beginning of the study?
   a) Yes (***one star***)
   b) No
   c) No description/ not clear

B. Comparability

1) Do main analyses of observational data control for confounders?
   a) Yes (***one star***)
   b) No
   c) Information not provided/ not clear

C. Outcome

1) Ascertainment of MHO/MetS in an obese cohort
   a) Secure record (e.g surgical records) (***one star***)
   b) Directly measured by the study(***one star***)
   c) Mixture of secure record and direct measurement by the study(***one star***)
   d) Self-report

e) No description/ not clear

2) Was follow-up long enough for outcomes to occur
    a) Yes (follow up exceeded 5 years) (***one star***)
    b) No
    c) No information given/ not clear

3) Adequacy of follow-up of cohorts
    a) Complete follow up- all subjects accounted for (***one star***)
    b) Subjects lost to follow-up unlikely to introduce bias- number lost less than or equal to 20% or description of those lost (suggested no different from those followed) (***one star***)
    c) Follow up rate less than 80% and no description of those lost
    d) No statement/ not clear
    e) Cross-sectional with retrospective report of exposures, thus not applicable

Thresholds for converting the Newcastle-Ottawa scales to AHRQ standards (good, fair, and poor):

Good quality: 3 or 4 stars in selection domain AND 1 star in comparability domain AND 2 or 3 stars in outcome/exposure domain

Fair quality: 2 stars in selection domain AND 1 star in comparability domain AND 2 or 3 stars in outcome/exposure domain

Poor quality: 0 or 1 star in selection domain OR 0 stars in comparability domain OR 0 or 1 stars in outcome/exposure domain
