# Supplementary material for: Life course factors associated with metabolically healthy obesity: a protocol for the systematic review of longitudinal studies
Source: Syst Rev. 2018 Mar 27;7:50. doi: 10.1186/s13643-018-0713-x (PMC5870377; doi:10.1186/s13643-018-0713-x)
Supplement: Supplementary file 6 — Quality assessment data extraction form. (PDF 231 kb) [file 13643_2018_713_MOESM6_ESM.pdf]

Additional file 6. Quality Assessment form

**Study ID**	**A.1**	**A.2**	**A.3**	**A.4**	**B.1**	**C.1**	**C.2**	**C.3**	**Score**
Citation details	Method of derivation of the cohort and details on the representativeness of the cohort of the source population.	Details on representativeness of the analysis sample of the full initial cohort.	Details on ascertainment of exposures.	Details on demonstration of presence/absence outcome at baseline.	Details on main analysis confounder adjustment.	Details on ascertainment of MHO/MetS in an obese group.	Details on follow-up duration of the study.	Details on the follow-up of the cohort.	Section A:<br>1)NS/(*)<br>2)NS/(*)<br>3)NS/(*)<br>4)NS/(*)<br>Section B:<br>1)NS/(*)<br>Section C:<br>1)NS/(*)<br>2)NS/(*)<br>3)NS/(*)
	**a)/b)/c)/d)/e) –NS/(*)**	**a)/b)/c) –NS/(*)**	**a)/b)/c)/d)/e)/f)/g) –NS/(*)**	**a)/b)/c) –NS/(*)**	**a)/b)/c) –NS/(*)**	**a)/b)/c)/d)/e) –NS/(*)**	**a)/b)/c) – NS/(*)**	**a)/b)/c)/d)/e) –NS/(*)**	**Quality: Poor/fair/Good**
